# Influence of bundled care treatment on functional outcome in patients with intracerebral hemorrhage

**DOI:** 10.3389/fneur.2024.1357815

**Published:** 2024-08-05

**Authors:** Anne Mrochen, Yu Song, Verena Harders, Jochen A. Sembill, Maximilian I. Sprügel, Stefan Hock, Stefan Lang, Tobias Engelhorn, Bernd Kallmünzer, Bastian Volbers, Joji B. Kuramatsu

**Affiliations:** ^1^Department of Neurology, Friedrich-Alexander-University Erlangen-Nürnberg (FAU), Erlangen, Germany; ^2^Department of Neurosurgery, The First Affiliated Hospital of Zhengzhou University, Zhengzhou, China; ^3^Department of Neuroradiology, Friedrich-Alexander-University Erlangen-Nürnberg (FAU), Erlangen, Germany

**Keywords:** ICH, bundle, treatment, PHE, HE

## Abstract

**Background and aims:**

General guideline recommendations in patients with intracerebral hemorrhage (ICH) include blood pressure-, temperature- and glucose management. The therapeutic effect of such a “care bundle” (blood pressure lowering, glycemic control, and treatment of pyrexia) on clinical outcomes becomes increasingly established. For the present study, we aimed to investigate associations of strict bundled care treatment (BCT) with clinical outcomes and characterize associations with key outcome effectors such as hematoma enlargement (HE) and peak perihemorrhagic edema (PHE).

**Methods:**

We screened consecutive ICH patients (*n* = 1,322) from the prospective UKER-ICH cohort study. BCT was defined as achieving and maintaining therapeutic ranges for systolic blood pressure (110–160 mmHg), glucose (80–180 mg/dL), and body temperature (35.5–37.5°C) over the first 72 h. The primary outcome was the functional outcome at 12 months (modified Rankin Scale (mRS) 0–3). Secondary outcomes included mortality at 12 months, the occurrence of hematoma enlargement, and the development of peak perihemorrhagic edema. Confounding was addressed by a doubly robust methodology to calculate the absolute treatment effect (ATE) and by calculating e-values.

**Results:**

A total of 681 patients remained for analysis, and 182 patients fulfilled all three BCT criteria and were compared to 499 controls. The ATE of BCT to achieve the primary outcome was 9.3%, 95% CI (1.7 to 16.9), *p* < 0.001; e-value: 3.1, CI (1.8). Mortality at 12 months was significantly reduced by BCT [ATE: −12.8%, 95% CI (−19.8 to −5.7), *p* < 0.001; e-value: 3.8, CI (2.2)], and no association was observed for HE or peak PHE. Significant drivers of BCT effect on the primary outcome were systolic blood pressure control (ATE: 15.9%) and maintenance of normothermia (ATE: 10.9%).

**Conclusion:**

Strict adherence to this “care bundle” over the first 72 h during acute hospital care in patients with ICH was independently associated with improved functional long-term outcome, driven by systolic blood pressure control and maintenance of normothermia. Our findings strongly warrant prospective validation to determine the generalizability especially in Western countries.

**Clinical trial registration:**ClinicalTrials.gov, identifier [ID: NCT03183167].

## Introduction

Intracerebral hemorrhage accounts for 11–22% of strokes and contributes to the burden of the disease with approximately 42% of the disability adjusted life-years due to the stroke (47 million life-years) (1, 2). Although the “one” breakthrough intervention improving functional outcome and mortality does not exist, a variety of treatment approaches possibly interacting with one another have been investigated (3). Baseline guideline-recommended interventions comprise an early and strict implementation of blood pressure, temperature, and glucose management (4). Over the last years, several large clinical trials and observational studies provided new evidence enhancing acute ICH care by investigating the potential benefit of single interventions (5–8). Nevertheless, the potential synergistic benefits of guideline-recommended treatments when combined remain elusive, especially in Western countries. Clustering interventions together as a care bundle lately revealed promising outcomes (9, 10). For example, Parry-Jones et al. focused on a “bundle” of treatments: reversal of coagulation status, referral to neurosurgery, blood pressure control, and admission to a neurological intensive care unit, and found a 6 to 12% absolute reduction in mortality (10). Most currently, the cluster-randomized INTERACT3 trial has been published demonstrating that implantation of a care bundle protocol in low- and middle-income countries without a previous standardized operating procedure for ICH patients resulted in improved functional outcome (11). This large trial (*n* = 7.036) included patients mainly from China and could document significant differences according to treatment allocation only for the parameter blood pressure, hence potentially driving the overall effect on the entire range of mRS estimates (common odds ratio 0.86; 95% CI: 0.76–0.97; *p* = 0·015).

The present study investigated whether the consistent and effective implementation of bundled care treatment (BCT) targets for systolic blood pressure, glucose levels, and temperature improved patient functional long-term outcomes after intracerebral hemorrhage. In addition, we aim to evaluate the key components of a care bundle and characterize associations with hematoma enlargement (HE) and peak perihemorrhagic edema (PHE).

## Methods

### Study participants and study design

**Figure 1:**
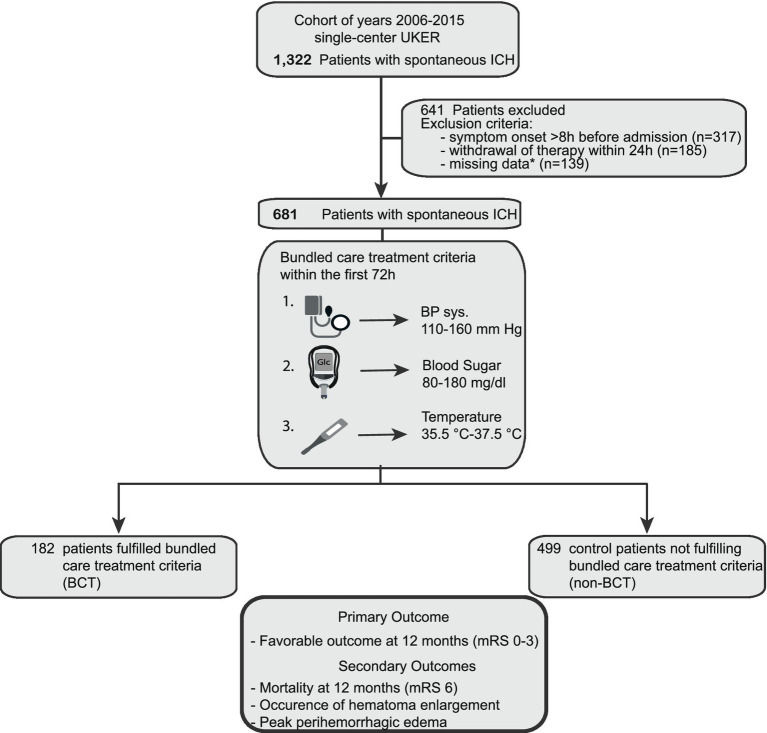
Flow chart of study participants. Overall, 1,322 patients with spontaneous ICH (years 2006–2015) were screened for analysis. 641 patients were excluded because of (i) symptom onset >8 h before admission (*n* = 317), (ii) withdrawal of therapy within the first 24 h (*n* = 185), and (iii) missing patient data (*n* = 139). 681 patients remained for primary and secondary outcome analyses. 182 fulfilled the bundle care treatment criteria, and 499 patients remained as controls. *Missing data consist of (i) more than 33.3% of maximum or minimum values of each BCT parameter over all 6-h intervals or (ii) follow-up imaging (dicom data for detailed analysis). BCT, bundled care treatment; ICH, intracerebral hemorrhage; UKER, Universitätsklinikum Erlangen Cohort of Patients With Spontaneous Intracerebral Hemorrhage.

We included patient data from the prospective single-center UKER-ICH registry [patients with spontaneous ICH; from 1 January 2006 until 31 December 2015 (NCT03183167)]. Detailed information and methods have been published previously (12–15). The study was approved by the local ethics committee and institutional review boards based on the central votes from Friedrich-Alexander-University Erlangen-Nuremberg, Germany (Re.No-4409 & 30_16B, 115_17B: “Retrospective analysis of patients with intracerebral haemorrhage,” approval date 20 June 2017) (12). Consent was obtained from patients or legal representatives. The procedures followed were in accordance with IRB ethical standards for human experimentation and the Helsinki Declaration of 1975. Patients with secondary ICH etiologies such as aneurysms, arteriovenous malformations, tumorous lesions, trauma, or coagulopathies other than oral anticoagulation were excluded (12–15). In addition, we excluded patients with symptom onset >8 h or withdrawal of therapy within 24 h according to previous studies (16) (Figure 1).

### Data acquisition

Data collection included baseline data on demographics (age and sex), prior comorbidities (hypertension, coronary artery disease, prior stroke, and abnormal kidney or liver function), prior medication (oral anticoagulation and antiplatelet medication), timing measures (time from symptom onset to first and second CT and time from symptom onset to treatment), and neurological status assessed using the National Institutes of Health Stroke Scale (NIHSS) upon hospital admission. In addition, the premorbid modified Rankin Scale was assessed (17). We evaluated hematoma characteristics (ICH location, intraventricular hemorrhage, and ICH volume) by neuroradiologists (S.H., S.L. and T.E.) blinded to clinical information. Assessment of mortality and functional outcome (modified Rankin Scale [mRS]) at 12 months was obtained by independent personnel, as previously reported (12). We recorded all available bundled care treatment (BCT) parameters, that is, measurement of systolic blood pressure, temperature, and blood sugar levels within the first 72 h.

### Definition of BCT

Patients with ICH were categorized into two groups: one group with patients fulfilling the BCT criteria over the first 72 h (BCT) and the control group of patients not fulfilling the criteria (non-BCT). The BCT criteria consisted of three target parameters: systolic blood pressure, temperature, and glucose management. We defined the following target ranges according to standard operating procedures at our institution in place over the study period: (1) systolic blood pressure between 110 mmHg and 160 mmHg, (2) temperature between 35.5°C and 37.5°C, and (3) blood sugar levels between 80 mg/dL and 180 mg/dL. We evaluated all available measurements, divided these into 6-h intervals, and recorded maximum and minimum values over each period for each BCT parameter. Patients were categorized into the BCT group, if less than 33.3% of maximum or minimum values of each interval (i.e., >4/12) were outside of the predefined range over all 6-h intervals. We selected the systolic blood pressure range of <160 mmHg according to an internal standard operating procedure at our institution in place before the publication of the INTERACT-II trial data (7). Since 2013 this has been modified to a target systolic level of <140 mmHg^7^. Body core temperature was measured using the tympanic or bladder temperature, and the chosen range was considered normothermia (35.5°C and 37.5°C) according to previous studies (5, 18, 19). Regarding blood sugar levels, we selected a conventional range of 80–180 mg/dL according to current AHA guideline which recommends treating hypo- and hyperglycemia to prevent adverse events (6, 20, 21).

### Primary and secondary outcomes

The primary outcome measure was the functional outcome at 12 months assessed by the ordinal modified Rankin Scale (mRS; 0: no deficit, through 5: severe disability and 6: death). Functional outcome was grouped into favorable (mRS 0–3) and unfavorable (mRS 4–6) outcomes, as previously described (8). Secondary outcomes compromised (i) mortality at 12 months, (ii) occurrence of hematoma enlargement defined as an ICH volume increase of more than 33% (relative) or 6 mL from initial to follow-up imaging, and (iii) peak perihemorrhagic edema (PHE). We assessed PHE on all available CT scans using a validated semi-automated threshold-based algorithm with a threshold range of 5–33 Hounsfield units (22). Peak PHE was defined as the maximum PHE volume measured during hospitalization and dichotomized according to the median split method (22).

### Statistical analysis

Statistical analyses were performed using STATA (Version 14·2) and R x64 3.2.0.^1^ In general, confounders were considered relevant using a standardized mean difference larger than 20%. To address confounding, we used adjusted odds ratios (OR) and a doubly robust confounder-adjusted methodology to calculate adjusted absolute treatment effects, that is, augmented inverse probability weighting (AIPW) (23). These adjustments were applied in two ways: (A) identified confounders associated with an increased propensity for BCT and (B) validated confounders associated with primary and secondary endpoints; confounders were identified based on standardized mean differences (SMD). For exploratory analysis of the primary outcome, we used the aforementioned adjustment methodology. The categorization of non-dichotomous variables was split by the 50th percentile. Interactions of exploratory subgroup analyses were analyzed by the subgroup-defining variable (variable×intervention) and were considered significant for a *p*-value of <0.05. Sensitivity analyses compromised the evaluation of unmeasured confounding (e-values) (24). The average data missingness was approximately 7% per patient without difference between BCT and non-BCT patients. Missing functional outcome information was handled by multiple imputations by conditional specifications after assessment of missingness (25).

## Results

### Study population

**Figure 2:**
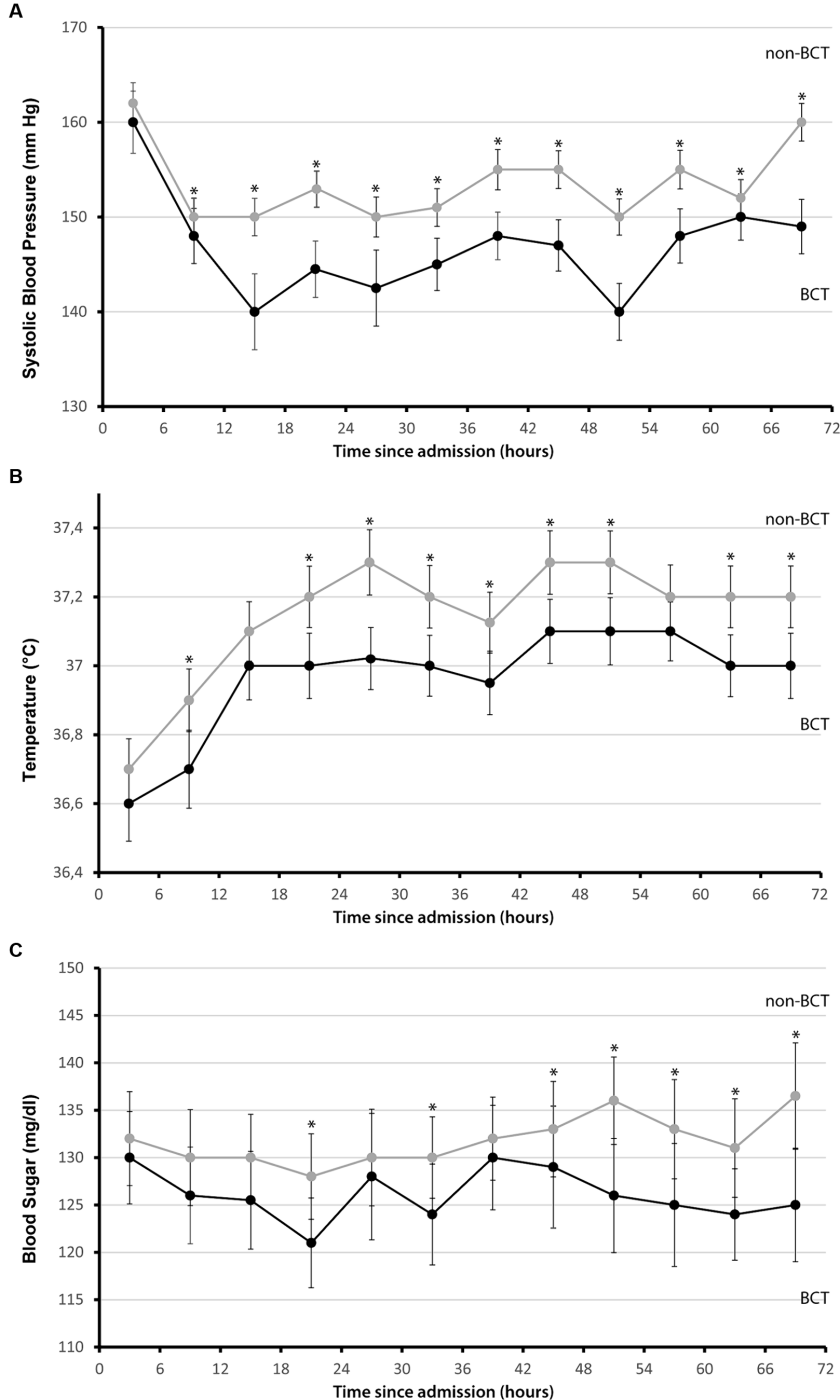
Systolic blood pressure, temperature, and glucose levels in 6-h intervals during the first 72 h mean values for **(A)** systolic blood pressure, mmHg, **(B)** temperature, °C, and **(C)** glucose levels, mg/dL measured in 6-h intervals since admission during the first 72 h separated for patients who fulfilled the bundle care treatment criteria (black) and for control patients (gray), maximum values were used. I bars indicated 95% confidence intervals. Asterisks mark significance *p ≤ 0.05.

We identified 681 patients from our prospective cohort study in spontaneous ICH (*n* = 1,322, Cohort of years 2006–2015 from UKER registry, Figure 1). A total of 182 patients (26.7%) fulfilled the strict BCT criteria and were compared to 499 controls (73.3%). We graphically displayed differences between these groups for all three BCT parameters over the first 72 h in Figure 2. Initial BCT measurements between groups were not significantly different (A, systolic blood pressure: 160 mmHg vs. 162 mmHg; B, temperature: 36.6°C vs. 36.7°C; blood sugar: 130 mg/dL vs. 132 mg/dL), and subsequent measurements over the 72-h time period significantly differed, that is, for systolic blood pressure in 92% (11/12, 6-h intervals), for temperature 75% (9/12, 6-h intervals), and for blood sugar levels 58% (7/12, 6-h intervals).

### Sensitivity analyses of confounders

Baseline characteristics are shown in Table 1. We identified significant imbalances in BCT patients than in non-BCT patients, that is, more frequent prior diagnosis of hypertension [absolute difference (AD) 6.1, 95% CI (1.2 to 11.1) %; SMD 0.19], less frequent treatment with external ventricular drainage [AD −7.6, 95% CI (−15.4 to 0.1) %; SMD −0.16], more frequent lobar ICH location [AD 7.9, 95% CI (−0.4 to 16.2) %; SMD 0.16], and less frequent intraventricular hemorrhage [AD −10.5, 95% CI (−18.8 to −2.3) %; SMD −0.21]. For evaluation of confounding regarding the primary endpoint, sensitivity analysis was dichotomized according to functional outcome (mRS 0–3 at 12 months, Table 2). The results show that patients with mRS 0–3 at 12 months were younger [AD 7.1, 95% CI (5.2 to 8.9), years, %; SMD 0.58], had lower premRS [AD 1, 95% CI (0.8 to 1.2), SMD 0.64], had less comorbidity of prior stroke/TIA [AD 9.7, 95% CI (4.0 to 15.4), %; SMD 0.25], lower ICH volumes [AD 14.2, 95% CI (10.5 to 17.8), mL; SMD 0.67], lower NIHSS values [AD 9, 95% CI (7.6 to 10.4), SMD 0.84], more frequent rate of intraventricular hemorrhage [IVH, AD 23.9, 95% CI (16.7 to 31.2), %; SMD 0.50], and less frequent prior use of oral anticoagulation [AD 7.7 (2.2 to 13.2), %; SMD 0.21]. We accordingly performed sensitivity analyses for secondary outcomes: mortality at 12 months, occurence of hematoma enlargement and peak perihemorrhagic edema, dichotomized according to median split method (value 25 cm^3^), for details please see Supplementary Tables S1–S3.

**Table 1:** Baseline characteristics: comparison of BCT patients vs. non-BCT patients.

ICH patients (*n* = 681)	Non-BCT (*n* = 499)	BCT (*n* = 182)	Absolute difference (95% CI)	SMD
Age, mean (SD), years	69.7 (12.6)	69.0 (12.5)	−0.7 (−2.9 to 1.4)	−0.06
Female sex, no. (%)	212 (42.5%)	81 (44.5%)	2.0 (−6.4 to 10.4)	0.04
Pre-stroke mRS, median (IQR)	0 (0–2)	1 (0–2)	1 (0.6 to 1.4)	0.01
Medical history, no. (%)				
Hypertension	430 (86.2%)	168 (92.3%)	6.1 (1.2 to 11.1)	0.19
Diabetes mellitus	139 (27.9%)	46 (25.3%)	−2.6 (−10.0 to 4.9)	−0.06
Liver dysfunction	37 (7.4%)	14 (7.7%)	0.3 (−4.2 to 4.8)	0.01
Kidney dysfunction	73 (14.6%)	24 (13.2%)	−1.4 (−7.3 to 4.4)	−0.04
Hypercholesterolemia	199 (39.9%)	79 (43.4%)	3.5 (−4.9 to 11.9)	0.07
Coronary artery disease	113 (22.7%)	37 (20.3%)	−2.3 (−9.2 to 4.6)	−0.06
Prior stroke/TIA	98 (19.6%)	30 (16.5%)	−3.2 (−9.6 to 3.3)	−0.08
Prior oral anticoagulation	80 (16.0%)	33 (18.1%)	2.1 (−4.4 to 8.6)	0.06
Antiplatelet use	140 (28.1%)	51 (28.0%)	−0.0 (−7.7 to 7.6)	−0.00
Neurological status				
Glasgow Coma Scale^a^, median (IQR)	13 (9–15)	13 (10–15)	0 (−0.8 to 0.8)	0.05
NIHSS^b^, median (IQR)	13 (6–20)	13 (7–19)	0 (−2.0 to 2.0)	0.04
Max-ICH score^c^, median (IQR)	4 (2–5)	4 (2–5)	0 (−0.7 to 0.7)	−0.07
Diagnostic imaging				
IVH, no. (%)	239 (47.9%)	68 (37.4%)	−10.5 (−18.8 to −2.3)	−0.21
Lobar ICH location, no. (%)	169 (33.9%)	76 (41.8%)	7.9 (−0.4 to 16.2)	0.16
Deep ICH location, no. (%)	259 (51.9%)	84 (46.2%)	−5.7 (−14.2 to 2.7)	−0.11
Infratentorial ICH location, no. (%)	68 (13.6%)	19 (10.4%)	−3.2 (−8.6 to 2.2)	−0.10
ICH volume, median (IQR), cm^3^	13.5 (5.1–35.8)	15.8 (5.3–32.4)	2.4 (−2.1 to 6.8)	0.00
Follow-up imaging				
Hematoma enlargement^d^, no. (%)	118 (23.7%)	45 (24.7%)	1.0 (−6.2 to 8.4)	0.03
Peak perihemorrhagic edema^e^, median (IQR)	23.9 (9.7–48.1)	27.5 (9.8–49.8)	3.6 (−2.5 to 9.8)	0.07
Time windows				
Time onset to arrival, median (IQR), min	74 (28–224)	109 (46–208)	35 (−8.1 to 78.1)	0.09
Time first to second CT, median (IQR), h	21 (12–29)	20 (13–27)	−1 (−3.7 to 1.1)	−0.04
Inhospital measures				
EVD, no. (%)	178 (35.7%)	51 (28.0%)	−7.6 (−15.4 to 0.1)	−0.16
Surgical intervention, no (%)	40 (8.0%)	17 (9.3%)	1.3 (−3.5 to 5.1)	0.05
Ventilation, no (%)	231 (46.3%)	73 (40.1%)	−6.2 (−14.5 to 2.2)	−0.12
Ventilation duration, median (IQR), days	10 (3–21)	11 (3–19)	1 (−2.8 to 4.8)	−0.10

Absolute differences are presented in percent for frequency data and for scales or continuous variables as absolute differences according to the measurement unit (negative values indicate a decreased frequency or unit of measurement from the reference, that is, non-BCT). Standardized mean differences (SMDs) are presented to compare BCT patients vs. non-BCT patients.

BCT, bundled care treatment; CI, confidence interval; EVD, extraventricular drainage; ICH, intracerebral hemorrhage; IQR, Interquartile range; IVH, intraventricular hemorrhage; mRS, modified Rankin Scale, 0 no deficit to 6 death; no, number of patients; SD, standard deviation; SMD, standardized mean differences; TIA, transient ischemic attack.

a Glasgow Coma Scale (ranging from 3, comatose, to 15, alert).

b NIHSS, National Institutes of Health Stroke Scale (ranging from 0, no deficit, −40, severe neurological deficit; 40 is the maximum because in comatose ataxia is not scored).

c ICH score (ranging from 0 to 6, with higher scores indicating greater disability or fatal outcome (mRS 6) after ICH).

d Hematoma enlargement defined as an ICH volume increase of more than 33% (relative) or 6 mL from initial to follow-up imaging.

e Peak perihemorrhagic edema dichotomized according to median split (≥25 cm^3^).

**Table 2:** Baseline characteristics: comparison of patients who achieved the primary outcome (mRS 0–3) at 12 months vs. those who did not.

ICH patients (*n* = 681)	mRS 0–3 at 12 months (*n* = 288)	mRS 4–6 at 12 months (*n* = 393)	Absolute difference (95% CI)	SMD
Age, mean (SD), years	65.4 (12.5%)	72.5 (11.8%)	7.1 (5.2 to 8.9)	0.58
Female sex, no. (%)	119 (41.3%)	174 (44.3%)	3.0 (−4.6 to 10.5)	0.06
Pre-stroke mRS, median (IQR)	0 (0–1)	1 (0–2)	1 (0.8 to 1.2)	0.64
Medical history, no. (%)				
Hypertension	255 (88.5%)	343 (87.3%)	−1.3 (−6.2 to 3.7)	−0.04
Diabetes mellitus	83 (28.8%)	102 (26.0%)	−2.9 (−9.7 to 3.9)	−0.06
Liver dysfunction	18 (6.3%)	33 (8.4%)	2.1 (−1.8 to 6.0)	0.08
Kidney dysfunction	31 (10.8%)	66 (16.8%)	6.0 (0.9 to 11.2)	0.18
Hypercholesterolemia	140 (48.6%)	138 (35.1%)	−13.5 (−21.0 to −6.0)	−0.28
Coronary artery disease	57 (19.8%)	93 (23.7%)	3.9 (−2.4 to 10.1)	0.09
Prior stroke/TIA	38 (13.2%)	90 (22.9%)	9.7 (4.0 to 15.4)	0.25
Prior oral anticoagulation	35 (12.2%)	78 (19.9%)	7.7 (2.2 to 13.2)	0.21
Antiplatelet use	69 (24.0%)	122 (31.0%)	7.1 (0.4 to 13.8)	0.16
Neurological status				
Glasgow Coma Scale^a^, median (IQR)	14 (12–15)	12 (6–14)	−2 (−2.7 to −1.3)	−0.67
NIHSS^b^, median (IQR)	7 (4–14)	16 (10–25)	9 (7.6 to 10.4)	0.84
Max-ICH score^c^, median (IQR)	2 (1–4)	5 (4–6)	3 (2.7 to 3.3)	1.32
Diagnostic imaging				
IVH, no. (%)	90 (31.3%)	217 (55.2%)	23.9 (16.7 to 31.2)	0.50
Lobar ICH location, no. (%)	105 (36.5%)	140 (35.6%)	−0.8 (−8.1 to 6.5)	−0.02
Deep ICH location, no. (%)	136 (47.2%)	207 (52.7%)	5.4 (−2.1 to 13.0)	0.11
Infratentorial ICH location, no. (%)	44 (15.3%)	43 (10.9%)	−4.3 (−9.5 to 0.8)	−0.13
ICH volume, median (IQR), cm^3^	7.5 (2.3–17.9)	21.7 (8.6–45.1)	14.2 (10.5 to 17.8)	0.67
Follow-up imaging				
Hematoma enlargement^d^, no (%)	51 (17.7%)	112 (28.5%)	10.8 (4.5 to 17.1)	−0.24
Peak perihemorrhagic edema^e^, median (IQR)	14.8 (5.9–31.1)	34.6 (17.0–60.9)	19.9 (14.5 to 25.3)	0.64
Time windows				
Time onset to arrival, median (IQR), min	59 (30–181)	117 (29–247)	58 (22.1 to 93.6)	0.15
Time first to second CT, median (IQR), h	21 (14–33)	20 (12–27)	−1 (−3.6 to 0.6)	−0.22
Inhospital measures				
EVD, no. (%)	66 (22.9%)	163 (41.5%)	18.6 (11.7 to 25.4)	0.40
Surgical intervention, no (%)	21 (7.3%)	36 (9.2%)	1.8 (−2.3 to 6.0)	0.07
Ventilation, no (%)	78 (27.1%)	226 (57.5%)	30.4 (23.3 to 37.5)	0.64
Ventilation duration, median (IQR), days	8 (2–15)	11 (4–22)	3 (−0.9 to 6.9)	0.40

Absolute differences are presented in percent for frequency data and for scales or continuous variables as absolute differences according to the measurement unit (negative values indicate a decreased frequency or unit of measurement from the reference, that is, patients who achieved mRS 0–3). Standardized mean differences (SMD) are presented to compare patients who achieved the primary outcome vs those patients who did not.

CI, confidence interval; EVD, extraventricular drainage; ICH, intracerebral hemorrhage; IQR, Interquartile range; IVH, intraventricular hemorrhage; mRS, modified Rankin Scale, 0 no deficit to 6 death; no, number of patients; SD, standard deviation; SMD, standardized mean differences; TIA, transient ischemic attack.

a Glasgow Coma Scale (ranging from 3, comatose, to 15, alert).

b NIHSS, National Institutes of Health Stroke Scale (ranging from 0, no deficit, −40, severe neurological deficit; 40 is the maximum because in comatose ataxia is not scored).

c ICH score (ranging from 0 to 6, with higher scores indicating greater disability or fatal outcome (mRS 6) after ICH).

d Hematoma enlargement defined as an ICH volume increase of more than 33% (relative) or 6 mL from initial to follow-up imaging.

e Peak perihemorrhagic edema dichotomized according to median split (≥25 cm^3^).

### Analyses of the primary and secondary outcomes

**Figure 3:**
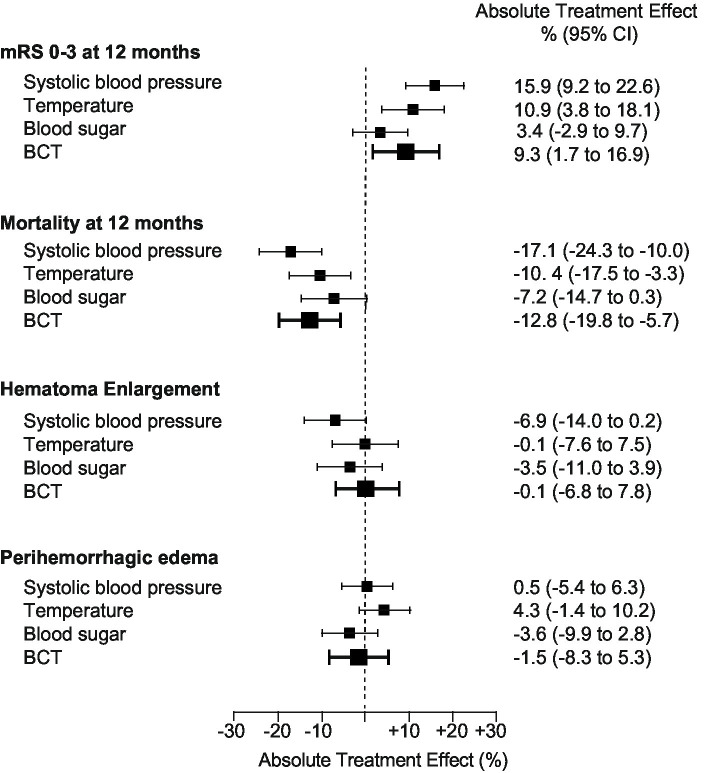
Associations of single BCT components to the absolute treatment effect. Results for the primary (modified Rankin Scale score 0–3 at 12 months) and secondary outcomes (mortality at 12 months), occurrence of hematoma enlargement, volume of peak perihemorrhagic edema, dichotomized according to median split (<25 cm^3^) are shown as adjusted absolute treatment effects separated for each target parameter: systolic blood pressure, temperature, and blood sugar, as well as BCT effect for categorized outcomes (bold), *n* = 681. Abbreviations/scores: BCT: bundled care treatment; hematoma enlargement was defined as an ICH volume increase of more than 33% (relative) or 6 mL from initial to follow-up imaging; peak perihemorrhagic edema was defined as maximum perihemorrhagic edema volume measured during hospitalization and dichotomized according to the median split method.

The adjusted absolute treatment effect (ATE) of BCT to achieve a favorable functional outcome at 12 months was 9.3%, 95% CI (1.7 to 16.9), *p* < 0·001 [adjusted OR, 1.86 (95% CI, 1.23–2.83), *p* < 0.005; (e-value, point estimate, 3.1, CI, 1.8)]. Among secondary outcomes, only mortality at 12 months was significantly reduced with an absolute treatment effect for BCT [ATE −12.8%, 95% CI (−19.8 to −5.7), *p* < 0.001; adjusted OR, 2.22 (95% CI, 1.40–3.53), *p* < 0.005; (e-value, point estimate, 3.8, CI, 2.2)]. There was no significant association of BCT regarding the occurrence of hematoma enlargement or peak perihemorrhagic edema [HE: ATE −0.1%, 95% CI (−6.8 to 7.8), *p* = 0.89; PHE: ATE −1.5%, 95% CI (−8.3 to 5.3, *p* = 0.65)] (Figure 3).

### Exploratory subgroup analyses

**Figure 4:**
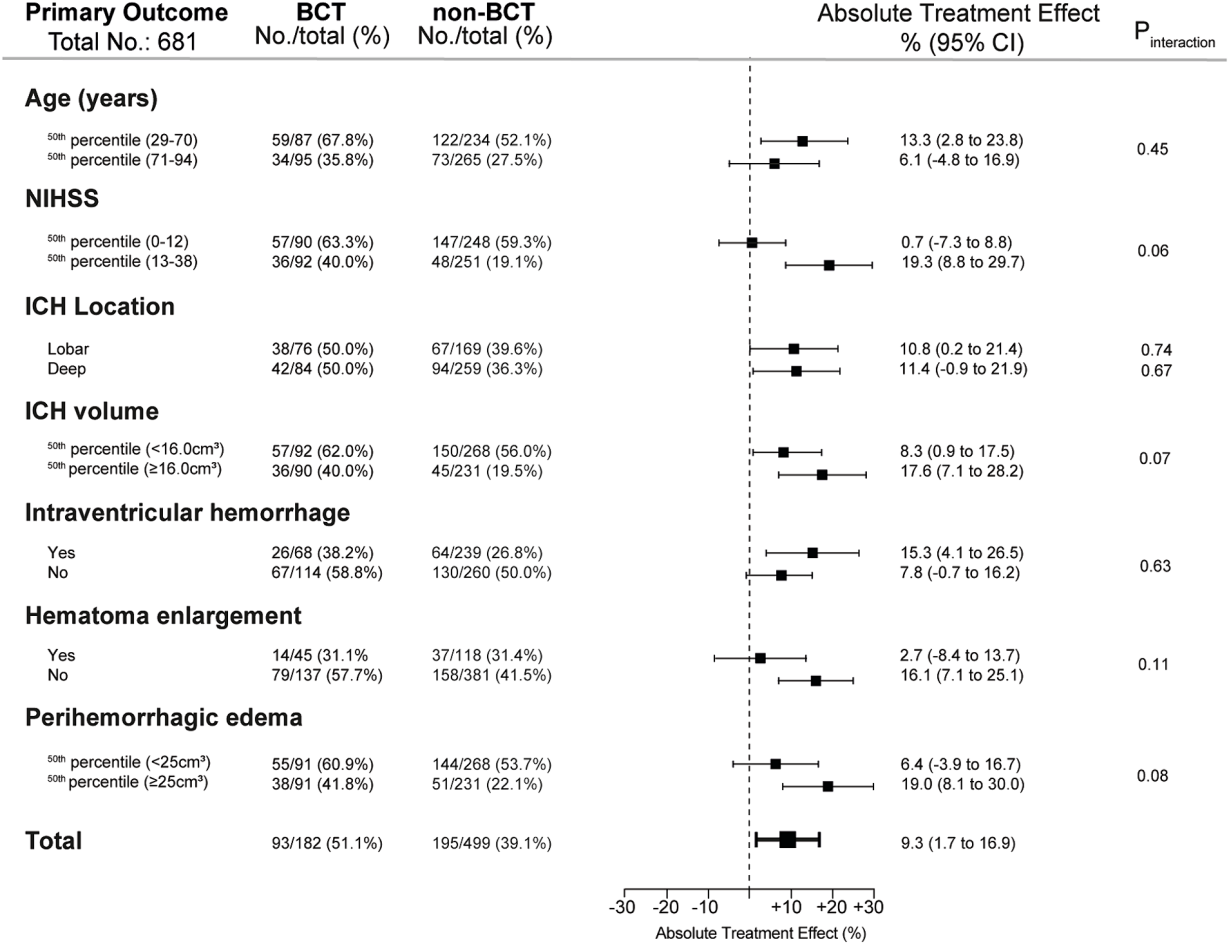
Exploratory subgroup analyses of the primary outcome. The results for the primary outcome (modified Rankin Scale score 0–3 at 12 months) are shown as crude frequency data and adjusted absolute treatment effects (*n* = 681). Interactions of exploratory subgroup analyses were analyzed by the subgroup-defining variable (variable×intervention) and were considered significant for *p* < 0.05. Abbreviations/scores: ICH: intracerebral hemorrhage; ICH volume was dichotomized according to median split (≥16.0 cm^3^). NIHSS, National Institutes of Health Stroke Scale. Hematoma enlargement defined as an ICH volume increase of more than 33% (relative) or 6 mL from initial to follow-up imaging; peak perihemorrhagic edema was defined as maximum perihemorrhagic edema volume measured during hospitalization and dichotomized according to the median split method (≥25.0cm^3^).

For associations of BCT with the primary outcome according to predefined subgroups (Figure 4), significant ATE was found in younger patients aged 29–70 years, ATE 13.3%, 95% CI (2.8 to 23.8), in patients with higher NIHSS values, ATE 19.3%, 95% CI (8.8 to 29.7), in patients with larger ICH volumes (≥16.0 cm^3^), ATE 17.6%, 95% CI (7.1 to 28.2) and larger peak edema volumes (≥25.0 cm^3^), ATE 19.0%, 95% CI (8.1 to 30.0). In addition, significant ATE was found in patients with intraventricular hemorrhage, ATE 15.3%, 95% CI (4.1 to 26.5), and for patients without hematoma enlargement, ATE 16.1%, 95% CI (7.1 to 25.1). Significant interactions between treatment and subgroup categories were not detected (all *p* > 0.05).

### Contribution of the components of BCT to the overall treatment

To assess the contribution of each component of BCT to the overall effect, we investigated the treatment effect separated for each component, that is, glucose, blood sugar, and systolic blood pressure management (Figure 3). Therefore, ICH patients were categorized according to the above-mentioned target parameters (see also Methods). Accordingly, we addressed confounding by sensitivity analyses and doubly robust methodology as aforementioned. Among the three components, blood sugar management revealed no significant treatment effects for primary and secondary endpoints. Both systolic blood pressure and temperature management revealed a significant absolute treatment effect regarding the primary endpoint and mortality at 12 months [BP: mRS 0–3: ATE 15.9%, 95% CI (9.2 to 22.6), *p* < 0.001; mRS 6: ATE −17.1%, 95% CI (−24.3 to −10.0), *p* < 0.001; temperature: mRS 0–3: ATE 10.9%, 95% CI (3.8 to 18.1), *p* < 0.002; mRS 6: ATE −10.4%, 95% CI (−17.5 to −3.3), *p* < 0.001]. Regarding the occurrence of hematoma enlargement, only systolic blood pressure showed a trend toward a treatment effect [ATE −6.9%, 95% CI (−14.0 to 0.2), *p* = 0.06]. Neither overall BCT, nor the separated components revealed a significant treatment effect on peak PHE.

## Discussion

As key findings, we observed that a “care bundle” consisting of strict control of systolic blood pressure lowering, glucose levels, and normothermia over 72 h was related to improved functional long-term outcomes and survival in patients with intracerebral hemorrhage. Specifically, BCT showed larger treatment effects in younger but more severely affected ICH patients. Furthermore, we could identify that especially systolic blood pressure control and maintenance of normothermia contributed to the effectiveness of BCT. The questions arise, what may be the underlying mechanisms for this treatment effect of BCT and what is the contribution of each component to this overall effect?

Within analysis of the single components of BCT, we confirmed that lowering blood pressure contributed to a reduction of hematoma enlargement which consequently translated to improved functional outcomes. These results are, with respect to previous studies, not surprising (12, 26–28). Guidelines recommend a careful titration of blood pressure lowering to ensure continuous smooth and sustained control, avoiding peaks and large variability in SBP based on INTERACT-2 and Antihypertensive Treatment of Acute Cerebral Hemorrhage 2 (ATACH-2) for early intensive BP lowering (7, 8). It is important to note that blood pressure reduction also carries potential risks as highlighted by the ATACH-2 trial (8). Nevertheless, early treatment of blood pressure may reduce the risk of hematoma enlargement and improve functional outcomes which is supported by our analyses.

More interestingly, also maintenance of normothermia contributed to a significant improvement in functional outcomes which is controversially debated (5, 19, 29–31). Possible detrimental mechanisms of fever are hypothesized to contribute to the development of peak PHE (18, 31–33). In addition, in patients who develop hyperthermia, underlying infections may be present, which can negatively impact their outcomes. Nevertheless, we did not observe a statistically significant association with maintenance of normothermia and larger peak PHE, but we observed a statistical trend, ATE: 4.3 (−1.4 to 10.2) deserving further more in-depth investigations. Theoretically, this potential effect may vary over time, may be associated with the interventional time window (0–72 h), and may differ according to the time course of peak PHE development (34). Importantly, early PHE within 72 h has been reported as a predictor of peak PHE volume (31).

Targeted glucose management as the third part of our treatment bundle did not reveal any significant treatment effect. It is recommended that either hyperglycemia or hypoglycemia should be treated to prevent adverse events that may worsen outcomes (20, 21, 35–37). Previous studies do not indicate that strict targeted glucose management leads to improved outcomes in patients with ICH, which is also not supported by our results. In general, episodes with severe hypo- or hyperglycemia should be avoided during intensive care treatment, and randomized controlled data provided that severe hypoglycemic episodes were independently associated with increased mortality, yet without causal inference (6). From our data, we did not observe more frequent severe hypoglycemia episodes (<60 mg/dL) within the measured 72-h interval between both groups [2/182 (1%) BCT vs. 5/499 (1%) non-BCT; data not shown]. However, the extent to which strict implementation of specific glucose target values in the treatment of intracerebral hemorrhage affects clinical outcomes is still controversial.

Consequently, lowering blood pressure and managing temperature are substantial components of BCT to improve outcomes in intracerebral hemorrhage. Even if glucose management seems to have an inferior role, it is essential to prevent hypo- or hyperglycemia events to prevent adverse events.

However, the recently published cluster-randomized INTERACT3 trial revealed that implementing a novel care bundle protocol (early intensive blood pressure lowering, management algorithms for hyperglycemia, pyrexia, and abnormal anticoagulation) at hospitals without a prior standardized operating procedure improves functional outcome (11). The trial was undertaken mainly in low- and middle-income countries (contributing >99% of patients), and the authors concluded that the incorporation of such a care bundle protocol resulted in improved functional outcomes and assumed that “the overall treatment effect seems to have been driven by intensive blood pressure lowering” (11). Importantly, the parameters of temperature and glucose management did not show significant inter-group differences, and therefore, the question of whether these parameters contribute to improved outcomes remains unanswered. Our study gives initial insights into the relevance of the individual bundle components and identifies blood pressure lowering and temperature management as drivers of this overall treatment effect. In addition, key outcome effectors such as hematoma enlargement and perihemorrhagic edema development including inflammatory processes seemed to be influenced and theoretically may be considered as mechanistically relevant to functional outcome. This assumption is supported by our observation that patients with more severe intracerebral hemorrhages (ICHs) and a higher incidence of intraventricular hemorrhage experienced a greater treatment effect.

Thereby, our study revealed that beneficial associations of BCT may exist when strictly controlled even in a hospital in Europe with existing SOPs executed at a dedicated neurological intensive care unit. INTERACT3 dominantly included patients with deep hypertensive ICH (88%), which was the effect-driving subgroup, as compared to data of this study with only 50% of patients with deep ICH. Hence, we provide data on the beneficial associations with strict BCT including all other ICH locations as non-trial selected real-world cohort.

Limitations of this study include low patient numbers in the BCT group, especially for exploratory analyses, and limitations inherent to the retrospective nature of this cohort study. In addition, data from this monocentric cohort may not be generalizable, and exact dosing and frequency of therapeutic medications were not part of this investigation. We cannot fully exclude that patients who stayed spontaneously within target ranges suffered less from major cerebrovascular and hemodynamic disruption as a consequence of the ICH. Therefore, we addressed confounding such as premorbid status, age, and ICH severity by robust statistical methodologies and sensitivity analyses. Furthermore, within the investigated time span (2005–2015), the guideline recommendation for systolic blood pressure lowering has changed following the publication of the INTERACT-II trial data (7). After 2013, this has been modified to a target systolic level of <140 mmHg at our institution. We did not observe bias over these differing treatment periods, that is, patients treated between 2013 and 2015 comprised 28% (*n* = 191/681) of the entire cohort, of which 25% (*n* = 46/182) were grouped into BCT compared to 29% (*n* = 145/499) grouped into non-BCT (data not shown). Potentially, we are missing stronger effects of more stringent blood pressure management due to the low proportion achieving less than 140 mmHg systolic according to current guideline recommendations. Bias due to confounding including also confounding by indication, residual bias, and unmeasured confounding cannot be fully ruled out but were addressed by robust statistical methodologies and sensitivity analyses.

## Conclusion

Strict and consequent adherence to BCT consisting of systolic blood pressure lowering, treatment of pyrexia, and glucose management in patients with intracerebral hemorrhage was associated with improved functional outcomes at 12 months, specifically in younger patients with larger ICH volumes and larger edema volumes. Furthermore, we could identify that especially blood pressure lowering and treatment of pyrexia contributed to the beneficial associations of BCT whereas management of blood sugar seemed to have an inferior role. Future prospective studies are warranted to validate the effects of BCT and its components.

## Data availability statement

The data that support the findings of this study are available from the corresponding author upon reasonable request.

## Ethics statement

The studies involving humans were approved by local ethics committee and institutional review boards based on the central votes from Friedrich-Alexander-University Erlangen-Nuremberg, Germany (Re.No-4409 & 30_16B, 115_17B: “Retrospective analysis of patients with intracerebral haemorrhage,” approval date 20th June 2017). The studies were conducted in accordance with the local legislation and institutional requirements. The participants provided their written informed consent to participate in this study.

## Author contributions

AM: Conceptualization, Formal analysis, Writing – original draft, Writing – review & editing, Project administration, Validation. YS: Data curation, Formal analysis, Writing – original draft, Writing – review & editing. VH: Data curation, Writing – review & editing. JS: Writing – review & editing. MS: Writing – review & editing. SH: Methodology, Writing – review & editing. SL: Methodology, Writing – review & editing. TE: Methodology, Writing – review & editing. BK: Writing – review & editing. BV: Methodology, Writing – review & editing. JK: Conceptualization, Project administration, Validation Writing – original draft, Writing – review & editing.

## Footnotes

^1^www.r-project.org
