# Supplementary material for: Influence of bundled care treatment on functional outcome in patients with intracerebral hemorrhage
Source: Front Neurol. 2024 Aug 5;15:1357815. doi: 10.3389/fneur.2024.1357815 (PMC11330843; doi:10.3389/fneur.2024.1357815)
Supplement: Supplementary file 1 [file Data_Sheet_1.PDF]

ICH patients (n=681)	mRS 0-5 at 12 months (n= 482)	mRS 6 at 12 months (n=199)	Absolute difference (95%CI)	SMD
Age, mean (SD), years	67.8 (12.5%)	73.6 (12.0%)	5.8 (3.7 to 7.8)	0.47
Female sex, No. (%)	206 (42.7%)	87 (43.7%)	0.9 (-7.2 to 9.2)	0.02
Pre-stroke mRS, median (IQR)	0 (0.1)	1 (0-2)	1 (0.7 to 1.3)	0.42
Medical History, No. (%)				
Hypertension	432 (89.6%)	166 (83.4%)	-6.2 (-12.1 to -0.4)	-0.17
Diabetes Mellitus	136 (28.2%)	49 (24.6%)	-3.6 (-10.8 to 3.6)	-0.08
Liver Dysfunction	37 (7.7%)	14 (7.0%)	-0.6 (-4.9 to 3.6)	-0.02
Kidney Dysfunction	57 (11.8%)	40 (20.1%)	8.3 (2.0 to 14.5)	0.23
Hypercholesterinemia	213 (44.2%)	65 (32.7%)	-11.5 (-19.4 to -3.6)	-0.24
Coronary artery disease	99 (20.5%)	51 (25.6%)	5.1 (-2.0 to 12.1)	0.12
Prior Stroke/TIA	76 (15.8%)	52 (26.1%)	10.4 (3.4 to 17.3)	0.26
Prior oral anticoagulation	60 (12.5%)	53 (26.6%)	14.2 (7.4 to 21.0)	0.36
Antiplatelet use	130 (27.0%)	61 (30.7%)	3.7 (-3.8 to 11.2)	0.08
Neurological status				
Glasgow Coma Scale[a], median (IQR)	14 (11-15)	11 (3-13)	-3 (-3.6 to -2.4)	-0.68
NIHSS[b], median (IQR)	10 (5-17)	17 (11-32)	7 (4.8 to 9.2)	0.67
Max-ICH score[c], median (IQR)	3 (2-5)	5 (4-6)	2 (1.6 to 2.4)	1.00
Diagnostic Imaging				
IVH, No. (%)	196 (40.7%)	111 (55.8%)	15.1 (6.9 to 23.3)	0.30
Lobar ICH location, No. (%)	172 (35.7%)	73 (36.7%)	1.0 (-7.0 to 8.9)	0.02
Deep ICH location, No. (%)	241 (50.0%)	102 (51.3%)	1.3 (-7.0 to 9.5)	0.03
Infratentorial ICH location, No. %	63 (13.1%)	24 (12.1%)	-1.0 (-6.4 to 4.4)	-0.03
ICH volume, median (IQR), cm3	10.5 (3.9-27.7)	24.0 (9.2-46.5)	13.5 (9.2 to 17.7)	0.48
Follow- up Imaging				
Hematoma enlargement[d], No (%)	98 (20.3%)	65 (32.7%)	12.3 (4.9 to 19.8)	-0.29
Peak perihemorrhagic edema[e], median (IQR)	20.0 (7.7 to 42.3)	38.3 (17.7-63.2)	18.6 (12.7 to 24.6)	0.37
Time windows				
Time onset to arrival, median (IQR), min	76 (30-224)	106 (29-215)	31 (-11.8 to 73.8)	-0.01
Time first to second CT, median (IQR), h	21 (14-28)	19 (11-28)	-2 (-3.8 to 8.3)	-0.11
Inhospital measures				
EVD, No. (%)	144 (29.9%)	85 (42.7%)	12.8 (4.8 to 20.8)	0.27
Ventilation, No (%)	181 (37.6%)	123 (61.8%)	24.3 (16.2 to 32.3)	0.50
Ventilation duration, median (IQR), days	13 (5-23)	9 (3-16)	-4 (-7.4 to -0.6)	-0.33

Supplemental Table 1. Baseline characteristics: Comparison of patients who achieved mRS 0-5 at 12 months vs those who did not. Absolute differences are presented in percent for frequency data and for scales or continuous variables as absolute differences according to the measurement unit (negative values indicate a decreased frequency or unit of measurement from the reference, i.e. patients with mRS 0-5). Standardized mean differences (SMD) are presented to compare patients who achieved the secondary outcome vs patients who did not.

Abbreviations: CI, confidence interval; EVD, extraventricular drainage; ICH, intracerebral haemorrhage; IQR, Interquartile range; IVH, intraventricular haemorrhage; mRS, modified Rankin Scale, 0 no deficit to 6 death; No, number of patients; SD, standard deviation; SMD, standardized mean differences; TIA, transient ischemic attack; a Glasgow Coma Scale (ranging from 3, comatose, to 15, alert) b NIHSS, National Institutes of Health Stroke Scale (ranging from 0, no deficit, –40, severe neurological deficit; 40 is the maximum because in comatose ataxia is not scored) c ICH score (ranging from 0 to 6, with higher scores indicating greater disability or fatal outcome (mRS 6) after ICH) d Hematoma enlargement defined as an ICH volume increase of more than 33% (relative) or 6ml from initial to follow-up imaging e Peak perhemorrhagic edema dichotomized according to median split (≥25cm³).

ICH patients (n=681)	Without hematoma enlargement[a] (n= 518)	With hematoma enlargement[a] (n= 163)	Absolute difference (95%CI)	SMD
Age, mean (SD), years	69.3 (12.6)	70.1 (12.7)	0.8 (-1.4 to 3.0)	0.06
Female sex, No. (%)	219 (42.3%)	74 (45.4%)	3.1 (-5.6 to 11.9)	0.06
Medical History, No. (%)				
Pre-stroke mRS	0 (0-2)	1 (0-2)	1 (0.6 to 1.4)	0.14
Hypertension	457 (88.2%)	141 (86.5%)	-1.7 (-7.7 to 4.2)	-0.05
Diabetes Mellitus	142 (27.4%)	43 (26.4%)	-1.0 (-8.8 to 6.7)	-0.02
Liver Dysfunction	32 (6.2%)	19 (11.7%)	5.5 (-0.1 to 10.8)	0.19
Kidney Dysfunction	75 (14.5%)	22 (13.5%)	-1.0 (-7.0 to 5.1)	-0.03
Hypercholesterinemia	217 (41.9%)	61 (37.4%)	-4.4 (-13.0 to 4.1)	-0.09
Coronary artery disease	111 (21.4%)	39 (23.9%)	2.5 (-4.9 to 9.9)	0.06
Prior Stroke/TIA	96 (18.5%)	32 (19.6%)	1.1 (-5.9 to 8.1)	0.03
Prior oral anticoagulation	78 (15.1%)	35 (21.5%)	6.4 (-0.6 to 13.4)	0.17
Antiplatelet use	149 (28.8%)	42 (25.8%)	-3.0 (-10.8 to 4.8)	-0.07
Neurological status				
Glasgow Coma Scale[b], median (IQR)	13 (10-15)	13 (9-15)	0 (-0.9 to 0.9)	-0.09
NIHSS[c], median (IQR)	12 (5-19)	14 (7-21)	2 (-0.3 to 4.3)	0.15
Max-ICH score[d], median (IQR)	4 (2-5)	4 (3-5)	0 (-0.7 to 0.7)	0.19
Diagnostic Imaging				
IVH, No. (%)	248 (47.9%)	59 (36.2%)	-11.7 (-20.2 to -3.1)	-0.24
Lobar ICH location, No. (%)	167 (32.2%)	78 (47.9%)	15.6 (7.0 to 24.3)	0.32
Deep ICH location, No. (%)	276 (53.3%)	67 (41.1%)	-12.2 (-20.9 to -3.5)	-0.25
Infratentorial ICH location, No. (%)	69 (13.3%)	18 (11.0%)	-2.3 (-7.9 to 3.4)	-0.07
ICH volume, median (IQR), cm3	13.1 (5.3 to 31.8)	15.7 (4.1-41.8)	2.5 (-2.0 to 6.9)	0.09
Follow- up Imaging				
Peak perihemorrhagic edema[e], median (IQR)	21.6 (9.0 to 42.2)	40.0 (12.5-69.3)	18.4 (11.4 to 24.5)	0.41
Time windows				
Time onset to arrival, median (IQR), min	71 (30-210)	120 (28-250)	49 (4.4 to 93.6)	0.08
Time first to second CT, median (IQR), h	21 (13-29)	19 (11-28)	-2.0 (-4.3 to 0.8)	-0.14
Inhospital measures				
EVD, No. (%)	171 (33.0%)	58 (35.6%)	2.6 (-5.8 to 11.0)	0.05
Ventilation, No (%)	219 (42.3%)	85 (52.2%)	9.9 (1.1 o 18.6)	0.19
Ventilation duration, median (IQR), days	11 (4-21)	9 (2-18)	-2 (-5.7 to 1.7)	-0.23

Supplemental Table 2. Baseline characteristics: Comparison of patients with hematoma enlargement (defined as >6ml or 33% (relative) from initial to follow-up imaging) vs without hematoma enlargement.

Absolute differences are presented in percent for frequency data and for scales or continuous variables as absolute differences according to the measurement unit (negative values indicate a decreased frequency or unit of measurement from the reference, i.e. patients without hematoma enlargement). Standardized mean differences (SMD) are presented to compare patients who achieved the secondary outcome vs patients who did not.

Abbreviations: CI, confidence interval; EVD, extraventricular drainage; ICH, intracerebral haemorrhage; IQR, Interquartile range; IVH, intraventricular haemorrhage; mRS, modified Rankin Scale, 0 no deficit to 6 death; No, number of patients; SD, standard deviation; SMD, standardized mean differences; TIA, transient ischemic attack; a Hematoma enlargement defined as an ICH volume increase of more than 33% (relative) or 6ml from initial to follow-up imaging b Glasgow Coma Scale (ranging from 3, comatose, to 15, alert). c NIHSS, National Institutes of Health Stroke Scale (ranging from 0, no deficit, –40, severe neurological deficit; 40 is the maximum because in comatose ataxia is not scored) d ICH score (ranging from 0 to 6, with higher scores indicating greater disability or fatal outcome (mRS 6) after ICH) e Peak perihemorrhagic edema dichotomized according to median split (≥25cm^3).

ICH patients (n=681)	Peak perihemorrhagic Edema[a] <25cm³ (n= 359)	Peak perihemorrhagic Edema[a] ≥25cm³ (n= 322)	Absolute difference (95%CI)	SMD
Age, mean (SD), years	69.9 (12.4)	69.0 (12.8)	-0.9 (-2.8 to 1.1)	-0.07
Female sex, No. (%)	157 (43.7%)	136 (42.2%)	-1.5 (-9.0 to 5.9)	-0.03
Pre-stroke mRS	0 (0-2)	0 (0-2)	0 (-0.3 to 0.3)	-0.09
Medical History, No. (%)				
Hypertension	323 (90.0%)	275 (85.4%)	-4.6 (-9.5 to 0.4)	-0.14
Diabetes Mellitus	118 (32.9%)	67 (20.8%)	-12.1 (-18.6 to -5.5)	-0.27
Liver Dysfunction	17 (4.7%)	34 (10.6%)	5.8 (1.8 to 9.8)	0.22
Kidney Dysfunction	52 (14.5%)	45 (14.0%)	-0.5 (-5.8 to 4.7)	-0.01
Hypercholesterinemia	165 (46.0%)	113 (35.1%)	-10.9 (-18.2 to -3.5)	-0.22
Coronary artery disease	78 (21.7%)	72 (22.4%)	0.6 (-5.6 to 6.9)	0.02
Prior Stroke/TIA	76 (21.2%)	52 (16.2%)	-5.0 (-10.9 to 0.8)	-0.13
Prior oral anticoagulation	54 (15.0%)	59 (18.3%)	3.3 (-2.3 to 8.9)	0.09
Antiplatelet use	98 (27.3%)	93 (28.9%)	1.6 (-5.2 to 8.4)	0.04
Neurological status				
Glasgow Coma Scale[b], median (IQR)	14 (11-15)	12 (8-14)	-2 (-2.7 to -1.3)	0.61
NIHSS[c], median (IQR)	8 (4-15)	16 (11-24)	8 (6.5 to 9.5)	0.61
Max-ICH score[d], median (IQR)	3 (1-5)	5 (4-6)	2 (1.7 to 2.3)	0.73
Diagnostic Imaging				
IVH, No. (%)	137 (38.2%)	170 (52.8%)	14.6 (7.2 to 22.0)	0.30
Lobar ICH location, No. (%)	92 (25.6%)	153 (47.5%)	21.9 (14.8 to 29.0)	0.47
Deep ICH location, No. (%)	175 (48.8%)	168 (52.2%)	3.4 (-4.1 to 10.9)	0.07
Infratentorial ICH location, No. (%)	85 (23.7%)	2 (0.6%)	-23.1 (-27.5 to -18.6)	-0.75
ICH volume, median (IQR), cm3	5.5 (2.1-11.8)	32.4 (16.7 to 53.3)	26.9 (25.1 to 31.4)	1.33
Follow- up Imaging				
Hematoma enlargement[e], No. (%)	64 (17.8%)	99 (30.8%)	12.9 (6.5 to 19.3)	0.30
Time windows				
Time onset to arrival, median (IQR), min	88 (29-224)	89 (32-220)	1 (-36.9 to 38.9)	0.03
Time first to second CT, median (IQR), h	21 (14-31)	19 (12-27)	-2 (-4.2 to 0.4)	-0.15
Inhospital measures				
EVD, No. (%)	94 (26.2%)	135 (41.9%)	15.7 (8.7 to 22.8)	0.34
Ventilation, No (%)	117 (32.6%)	187 (58.6%)	25.5 (18.2 to 32.7)	0.53
Ventilation duration, median (IQR), days	7 (2-16)	13 (5-22)	6 (2.4 to 9.6)	0.37

Supplemental Table 3. Baseline characteristics: Comparison of patients with peak perihemorrhagic edema <25cm³ vs those with ≥25cm³.

Absolute differences are presented in percent for frequency data and for scales or continuous variables as absolute differences according to the measurement unit (negative values indicate a decreased frequency or unit of measurement from the reference, i.e patients with peak perihemorrhagic edema <25cm³). Standardized mean differences (SMD) are presented to compare patients who achieved the secondary outcome vs patients who did not.

Abbreviations: CI, confidence interval; EVD, extraventricular drainage; ICH, intracerebral haemorrhage; IQR, Interquartile range; IVH, intraventricular haemorrhage; mRS, modified Rankin Scale, 0 no deficit to 6 death; No, number of patients; SD, standard deviation; SMD, standardized mean differences; TIA, transient ischemic attack; a Peak perihemorrhagic edema dichotomized according to median split (≥25cm³). b Glasgow Coma Scale (ranging from 3, comatose, to 15, alert). c NIHSS, National Institutes of Health Stroke Scale (ranging from 0, no deficit, –40, severe neurological deficit; 40 is the maximum because in comatose ataxia is not scored) d ICH score (ranging from 0 to 6, with higher scores indicating greater disability or fatal outcome (mRS 6) after ICH) e Hematoma enlargement defined as an ICH volume increase of more than 33% (relative) or 6ml from initial to follow-up imaging.
